# The Influence of Poverty and Culture on the Transmission of Parasitic Infections in Rural Nicaraguan Villages

**DOI:** 10.1155/2012/478292

**Published:** 2012-08-15

**Authors:** Abraar Karan, Gretchen B. Chapman, Alison Galvani

**Affiliations:** ^1^Yale College, Yale University, 19 Tower Parkway, New Haven, CT 06511, USA; ^2^Department of Psychology, Rutgers University, Piscataway, NJ 08854-8020, USA; ^3^Department of Epidemiology & Public Health, Yale University School of Medicine, New Haven, CT 06510, USA

## Abstract

Intestinal parasitic infections cause one of the largest global burdens of disease. To identify possible areas for interventions, a structured questionnaire addressing knowledge, attitude, and practice regarding parasitic infections as well as the less studied role of culture and resource availability was presented to mothers of school-age children in rural communities around San Juan del Sur, Nicaragua. We determined that access to resources influenced knowledge, attitude, and behaviors that may be relevant to transmission of parasitic infections. For example, having access to a clinic and prior knowledge about parasites was positively correlated with the practice of having fencing for animals, having fewer barefoot children, and treating children for parasites. We also found that cultural beliefs may contribute to parasitic transmission. Manifestations of *machismo* culture and faith in traditional medicines conflicted with healthy practices. We identified significant cultural myths that prevented healthy behaviors, including the beliefs that cutting a child's nails can cause tetanus and that showering after a hot day caused sickness. The use of traditional medicine was positively correlated with the belief in these cultural myths. Our study demonstrates that the traditional knowledge, attitude, and practice model could benefit from including components that examine resource availability and culture.

## 1. Introduction

There is a great need for social science research to improve the treatment and control of neglected tropical diseases (NTDs) through framing illness within the complex societal and cultural contexts in which it exists [1]. Intestinal parasitic infections are on the world health organization's list of NTDs and are among the most widespread health maladies in the developing world [2]. Intestinal parasites can be categorized into protozoa and helminthes [2]. Common protozoa include *Entamoeba histolytica *and *Giardia lamblia *while common helminthes include *Ascaris lumbricoides *(roundworm)*, Trichuris trichiura *(whipworm), and *Necator americanus *(hookworm) [2]. Nearly 4.5 billion individuals are at risk of helminth infection alone, which represent more than 40% of the disease burden due to all tropical diseases excluding malaria [3].

Health outcomes of intestinal parasitic infections include malnutrition, growth stunting, intellectual retardation, and cognitive as well as educational deficiencies [3]. Recurrent childhood infections can result in increased susceptibility to liver fibrosis, bladder cancer, and kidney failure in adulthood [4]. The efficacy of the four most common drugs against helminth infections is limited, particularly for late-stage illness [5]. The mass treatment programs that have been initiated by the World Health Assembly resolution with the goal of targeting 75% of school-aged children and other risk groups by the year 2010 required elevated dose regimens that were infeasible using chemotherapy alone [3]. With resistance developing to the available drugs, high reinfection rates, difficulty in developing new drugs, the need for proper education and behavioral changes among at-risk populations to curtail parasite transmission and prevent re-infection cycles is urgent [3].

However, the future success of these nonmedical interventions will require further understanding of the way in which culture, development, and hygienic behaviors interact with one another in communities afflicted with parasites. Several predictors of infection have been noted in previous studies, including greater susceptibility of females, lack of toilets, nonworking status of parents, lower socioeconomic status, younger age, household crowding, lower level of education, religion, nonuse of footwear, defecation practices, pig ownership, and lack of access to clean water [6, 7]. Cultural norms may interfere with proper hygienic practices by altering perceptions of disease, and treatment interventions should be appropriately tailored to account for these [8]. For instance, the spread of *Opisthorchis viverrini, *a common food-born parasite in South East Asia, was directly linked to the cultural habit of eating raw and fermented fish dishes [9]. Furthermore, populations in developing countries often lack the knowledge and resources to follow hygienic practices or may not perceive any threat from parasitic infections [10]. In a recent study by Tanner et al. [11], it was found that both a household's hygienic behavior and the mother's reliance on ethnomedical knowledge were directly correlated with a child's probability of parasitic infection. While the few studies to date which have focused on culture, knowledge, and behavior with relation to parasitic diseases have done so by correlating the presence of illness with these social characteristics, there is a dearth of research on the way in which behaviors, knowledge, poverty, and cultural factors interact amongst one another. We sought to further understand how the factors which are known to be correlated with increased parasitic susceptibility, particularly culture, poverty, and health literacy, were themselves correlated. By understanding the interplay of these factors more closely, stronger behavioral and educational interventions may be developed to create effective antiparasite strategies.

The several rural districts around San Juan del Sur, Nicaragua, have been part of an ongoing anti-parasite campaign focused on the distribution of water filters by Newton-San Juan del Sur Sister City Project (an NGO which provides a city partnership between Newton, MA, and San Juan del Sur) that has proven ineffective in reducing parasitic prevalence. To analyze the inefficacy of this intervention and holistically analyze parasitic re-infection cycles with respect to the numerous afore-mentioned factors, we conducted questionnaire-based interviews in the communities. We specifically focused on the influence of resource availability and culture on behaviors that are known to affect parameter transmission. The standard approach to analyze public health problems includes a survey of participants' knowledge of a particular disease, their attitude toward the disease, their behaviors and practices that make them more or less susceptible to acquiring the disease. Our study highlights and attempts to lessen the gap in disease treatment that can be bridged through the use of social science research which takes into account the host community's physical and cultural infrastructures.

## 2. Materials and Methods

To determine the interplay of personal, social, and cultural trends associated with the transmission of parasitic infections, we performed a survey of mothers with school-aged children in ten rural communities around San Juan del Sur in the Rivas district of Nicaragua. These communities were part of a community-wide fecal examination study in 2006 conducted by the local medical clinic and a medical student working with Newton-San Juan del Sur which found an 80% prevalence rate of *Giardia lamblia*, *Entamoeba histolytica*, and *Ascaris lumbricoides* and confirmed contamination of common water sources, especially wells (unpublished data acquired through the Newton-San Juan NGO). A follow-up analysis by a medical school in Managua in 2008 confirmed parasitic prevalence had not significantly decreased after initiation of deworming by the San Juan del Sur medical clinic and the water filtration campaign (unpublished data acquired from the San Juan del Sur Medical Center).

The mother is the central figure with regards to household cleanliness and children's health in Nicaragua and Latin America [12]. Additionally, school-age children are the most susceptible to parasitic infections, representing over 50% of the current 300 million people who are critically ill from parasites [4]. Thus, we targeted mothers with school-age children, which in San Juan del Sur was determined to be children 10 years of age and younger through recommendations from the local NGO. During July and August of 2009, we interviewed a total of 213 households in 10 different communities spanning the entire region surrounding San Juan del Sur. These communities were selected because they had been part of the NGO water filter intervention as well as the initial and follow-up parasitic fecal examination studies aforementioned.

Interviews were conducted with a comprehensive, 47-item questionnaire (Appendix B). The structured questionnaire was based on data collected in a pilot study conducted by the first author (A. Karan) during June of 2009 using an open-ended interview format. For the pilot phase, keyinformant interviews were conducted in three of the surrounding communities with 15 households determined to have individuals who were particularly knowledgeable about community perceptions of health. These individuals were recommended by the local NGO and the medical clinic. Some of these individuals were* brigadistas*, community health workers who were involved with the NGO (Appendix A). The results of this data were openly coded via thematic content analysis and the research hypothesis emerged in a grounded theory fashion. The structured questionnaire intended to examine the resulting hypothesis that culture and poverty influenced knowledge, attitude, and practice with regard to parasitic transmission. Using a mixedmethod approach, we derived the structured questionnaire from our key informant interviews to assess our hypothesis quantitatively.

The structured questionnaire was designed to address demographics, access to resources, knowledge and attitude toward health and intestinal parasites, practice of risky behaviors, and local cultural beliefs regarding parasites. We inquired about access to basic resources necessary for proper hygiene to create a basic assessment of a household's poverty level and further understand how poverty influences the propensity of infection. Several questions were designed in a yes/no format while some required numerical answers. Three questions were open-ended, requiring participants to list from personal practice or beliefs the times when one washed his or her hands, the symptoms that parasites caused, and the ways that individuals acquire parasitic infections.

The rural culture depends on natural medicine for healing as lack of transportation and money can make hospital care inaccessible. Specific cultural questions involving traditional medicine were developed after focus group discussions with *curanderos* (general traditional healers), *sobadores *(bone healers), and *promotores* (traditional birth attendants) were held in June of 2009 by the first author (A. Karan).

The interviewers administering the questionnaires were chosen by the NGO and teachers in the San Juan del Sur public school and were chosen as responsible, well-liked members of their community who would be most capable of properly conducting the questionnaire according to the training that was administered by one of the authors (A. Karan). We sought to conduct this portion of the research in a bottom-up approach that utilized local residents so as to avoid biases that a foreign interviewer may incur. Roughly 2 to 3 interviewers conducted the interviews in each community and were members of the community to which they were assigned. Prior to the beginning of the study, the interviewers were trained together at the local public school with regards to reducing bias in questionnaire administration, proper probing techniques, and sensitivity to the interviewees' decision to participate or answer specific questions. Verbal informed consent which explained the purposes, benefits, and risks of the study was provided to each participant prior to beginning the questionnaire. IRB approval was obtained from the Human Subjects Committee of Yale University as well as by a review committee consisting of members of the Newton- San Juan del Sur NGO.

## 3. Results

 The results of the questionnaire were tabulated into percentages and numerical averages which display trends in this study population. Analysis of these trends suggests several unique relationships between knowledge, attitude, behaviors, poverty, and culture as they relate to parasitic infections. The results allow for interpretation of a more comprehensive approach to tackling the problem of intestinal parasitosis.

Many questions were not completed by all 213 participants because they were only asked if a previous question was in the affirmative or the negative. For instance, only if a participant had a well he or she was asked if the latrine was located uphill from the well. Furthermore, some questionnaires were missing information because interviewers were accidentally skipping questions as the questionnaire was spatially condensed on two sheets of paper. Interviewer error was noted for questions which investigated participants' previous knowledge regarding parasitic transmission and health outcomes. Other missing data was due to participants' inability to understand certain questions. We did not hear any reports of participants refusing to answer questions that they understood.

Spearman correlation coefficients and *P* values are presented in Tables 4–6 listing correlations among variables. We correctly hypothesized that items relating to poverty and culture would correlate with knowledge, attitude, and practice items that suggest an increased risk of parasite transmission.

### 3.1. Demographics

 Women had an average age of 29 years with 3.6 years of education. The low levels of education are reflective of the cultural norms in which women work at home rather than pursuing higher education. On average a household had two children and each child averaged 4.9 years of age.

### 3.2. Resource Availability

Table 1 outlines the presence of resources such as wells, water filters, cement floors, soap, latrines, medication, fencing for animals, and access to education regarding sanitation and parasites. We found that most households lacked fencing (81%) to keep animals from wandering freely in the yards, and many only had dirt floors (65%). While 51% of the households had wells on their properties, other community members were still exposed to potentially contaminated well water through use of neighboring or community wells. 86% of households had latrines which are thought to be major contaminators of running subterraneous water that collects in wells located downhill from the latrine pit. Family wells were more elevated than the latrine in just 45% of the households suggesting that structural planning is imperative. Although local health workers known as *brigadistas* are responsible for monitoring the communities each month, the average time since a previous visit was 76 days. 43% of households had never seen a *brigadista* or had not for over one year. 61% of households had previous education about parasites from either a health center or local health worker. The distance to the closest health center was on average 8.3 km and it had been an average of 46 days since any individual had been to a health center. While this may point to a potential access problem, a fairly high 88% of families had treated their children for parasites in the past.

### 3.3. Knowledge and Attitude about General Hygiene

Table 2 outlines the knowledge and attitude toward general hygiene. In most households, there was a sufficient understanding of basic hygienic principles. Nonetheless, 20% of women believed the dirt in their yards was free of infection. Furthermore, 87% of people in the communities still believed, their wells were collecting clean water.

Attitudes toward maintenance of good hygiene were explored through questions regarding water filters and other areas associated with transmission. 75% of participants who did not already have a filter installed indicated a desire for one. Nonetheless, 20% thought the filter did not actually work. Furthermore, 92% of participants felt they already had good hygiene in the house, an attitude which was not reflected in practice.

### 3.4. Knowledge about Parasites

The population's knowledge with regards to parasitic infections is listed in Table 2 as well. The pilot study results indicated that the terms parasites, worms, and ameba were used interchangeably. Of the study participants, most had heard of at least one of the three. Subjects were also asked about symptoms that parasites cause, with vomiting (81%), stomachache (80%), and diarrhea (78%) being the most commonly recalled. The least reported were difficulty in school (9%), anemia (19%), lack of energy (30%), stunted growth (32%), and death (38%).

### 3.5. Attitude and Practice regarding Parasites

Attitudes and practice toward general hygiene and parasitic infections are listed in Table 2. In our survey, most participants reported being “afraid” of parasites (91%). In terms of unhealthy practices, roughly half (51%) said that children walk barefoot outside at times, while 34% of households had barefoot children outside at the time of their interview. A majority said they used well water directly for washing vegetables (87%) and cooking food (88%). 19% of the population reported that young children often walked around without diapers. Agricultural practices were questioned as well, and 39% of participants grew vegetables on their own soil. With regards to hand washing, the least reported times were after cleaning the yard (54%), changing a baby's diaper (54%), and touching animals (59%). The pilot study revealed a local perception that everyone always has parasites in their body, and 92% of participants in the study agreed with this.

### 3.6. Cultural Factors

In Table 3, the cultural factors associated with parasitic prevalence are listed. 46% of husbands helped in household cleaning. Additionally, pilot analysis showed two significant cultural beliefs. The belief that cutting a child's nails can cause tetanus and other sicknesses was thought to be true by 15% of subjects. Another belief was that a cold shower after a hot day caused sickness with which 86% of subjects agreed. Furthermore, 10% of households consulted *curanderos *and 67% believed in the efficacy of natural anthelmintics, including garlic and parsley used by 77% and 62%, respectively.

### 3.7. **Simply Correlations**

The correlations between different variables indicate interesting individual and societal trends that through interpretation present new ways at looking at treatment and control of parasites.

### 3.8. Lack of Infrastructure and Resources

Access to certain resources strongly correlated with several healthier practices (Table 4). For example, people who had previous information about parasites were less likely to have free-roaming animals on properties, had a lower prevalence of children barefoot at the time of the interview, and greater levels of parasitic treatment for children. Similarly, receiving previous information had a negative correlation with the false belief that well water is clean. Furthermore, the closer a participant lived by a clinic, the more likely he or she was to have heard previous information regarding parasites, the more likely that the children had been treated for parasites, and the less likely that animals were running free on the property or that the house had a dirt floor. Also, the presence of a dirt floor in the house was negatively correlated with the education level of the mother. Furthermore, the number of days since a *brigadista* had visited played a pivotal role with regards to water filters. The greater number of days since a visit negatively correlated with wanting a filter, believing a filter actually works, and having a functional filter.

### 3.9. Knowledge, Attitude, and Practice

Knowledge, attitude, and behavioral practice factors also showed unique correlations (Table 5). False beliefs were positively inter-correlated. For example, the belief that yards are clean correlated with the belief that animals are clean as well. This suggests that the same participants had flawed notions of cleanliness. Those participants who claimed to have seen parasites in their yard also thought that parasites were animals, which can explain this misconception. 

Attitudes toward personal hygiene were related to having certain resources. For example, participants who felt they had good hygiene also had soap, latrines, and husbands who helped around the house, which may explain why they had this belief. However, those who felt they had good hygiene did not show greater levels of self-reported hand washing than those who did not feel they had good hygiene. Thus, resources do not necessarily translate into practice.

Poor behavioral practices among children were also investigated. The greater number of children in a household correlated with a greater number of barefoot children present at the time of interview. This may be because with a greater number of children it is more likely to see a barefoot child, or because it is more difficult for the mother to ensure each child is practicing healthy habits. Furthermore, households which had barefoot children also were more likely to report children sometimes not wearing diapers.

### 3.10. Cultural Aspects

The cultural aspects of rural life with regards to transmission are highly significant and an understudied topic. People who saw *curanderos*, the traditional healers, were more likely to use natural remedies such as mint, wormseed, garlic, and guava leaf (Table 6). The two main cultural beliefs that influence public health (cutting a child's nails and cold showers causing illness) were correlated with a belief in traditional medicines. Thus, those who believed in the efficacy of traditional medicine also embraced traditional cultural beliefs. Furthermore, those who believed that traditional medicines in general have healing qualities also believed that they can cure parasites as opposed to solely reducing symptoms. Also, believing that this type of medicine cured parasites positively correlated with whether a participant used the medicine at home. Lastly, the husband helping in the household correlated with fewer children being barefoot at the time of interview. This suggests that with the help of the father, overall practice may be improved among children.

Nonetheless, some expected correlations were not found. For example, hearing previous information about parasites did not correlate with a reduced misperception about parasites. This may be an area of cultural preconceptions that withstand the influence of outside information.

## 4. Discussion

In this study, we examined knowledge, attitude, practice, poverty as measured by resource availability, and cultural influences with regards to parasitic infections in order to comprehensively evaluate the multidimensional nature demanded by effective treatment approaches. Our findings indicate that the households in San Juan had limited resources, moderate levels of knowledge, poor practices, and cultural influences that contribute to re-infection cycles. To effectively design prevention strategies, our study reveals the interplay between these spheres with a focus on poverty and cultural factors.

### 4.1. Poverty

Many households reported children often playing outside barefoot. Lack of footwear is a known risk factor and combined with the presence of unfenced animals is a strong predictor for infections such as hookworm that are transmitted through the foot [7]. Fencing for animals is prohibitively expensive for most families and thus poverty exacerbates a possible avenue of transmission. Similarly, many houses were constructed with only dirt floors as tiling is also too expensive, meaning children were exposed to dirt within the house. This accentuates a lack of differentiation between wearing shoes outside and inside the house.

Furthermore, the system of potable water is of great concern with regard to helminth infections [3]. Wells are readily contaminated due to the system of waste collection which occurs in pit latrines [10, 13]. Latrines are dug several meters into the ground which provides a direct source of contamination for the subterraneous water flow that passes through the fecal infected soil into the wells. Most wells were found to be downstream from latrines, elevating the risk of contamination [14]. The infrastructural composition of this system is due to a lack of developed water systems which is another manifestation of poverty. While further studies must be done with regards to the depth of the wells and the confirmation of actual contamination, we believe this is an important area of potential risk.

Food production and processing has been another route through which parasitic contamination can occur [15]. Both protozoans and helminthes have been associated with food contamination, including *Giardia lamblia* and *Ascaris lumbricoides *[16, 17]. Additionally, three of the four most common intestinal parasites found on produce were *Ascaris lumbricoides*,* Entamoeba histolytica*,and* Giardia lamblia*, all three of which were the most common parasites found in San Juan [18]. Without fencing for animals, the risk for contact between vegetables and contaminated soil is high. Moreover, studies are emerging suggesting the possible zoonotic transmission of *Giardia lamblia* from common livestock to humans while the notion of *Ascaris* transmission between pigs and humans is still largely unclear but possible [19–22]. Recent studies have also indicated that hookworm transmission from domestic animals to humans is likely [23]. While we do not believe that zoonotic transmission is the major route of infection in San Juan, considerations for livestock control are important for general hygiene and possibly for the prevention of some of the major parasites in our study. Construction projects in homes to tile floors and create fenced areas for animals as well as education regarding parasitic transmission cycles could help begin the shift toward improved sanitary conditions.

Correlations suggested that poor health practices tended to be clustered together in the same households and particularly larger sized households. Research has shown that poorer households tend to have greater numbers of children, particularly because children can serve as a labor source and provide additional income [24]. In this population, poor households who had more children, perhaps for child labor or economic necessity, were more likely to have poor health practices. The creation of incentivized programs to decrease the number of children born to each family, such as providing subsidized education that can allow a greater human capital investment in fewer children, will ultimately help to curtail parasitic transmission through creating a more educated populace and reducing poverty in households.

### 4.2. Cultural Beliefs

Culture has been shown to be closely intertwined with behaviors and health outcomes. Thus, we also examined cultural beliefs to gain a better understanding of parasites in the local context. Previous reports have shown differing attitudes toward parasitic infection in various cultures. For example, some indigenous African villagers believed worms to be a normal part of life and even health-promoting [25]. In this population, a commonly held belief was that everyone always has parasites. This belief suggests that the problem is one that people have accepted as regular and commonplace, perhaps mitigating the perceived risk associated with infection. While there was a belief that parasites are harmful for health, the extent to how much this actually influences behaviors is apparent in the recurrence of infection via avenues that are controllable. Furthermore, pilot analysis and general observation indicated that most households had shoes for all family members but children were often permitted to play barefoot. Yet, because there was a high knowledge regarding parasite transmission cycles and resources were available, attitude and ultimately culture may be responsible for the continued poor behaviors. Similar observations have been made in other helminth-infected communities in which nonuse of available resources was due to personal choice [26].

Reliance on traditional healers has been shown to interfere with modern healthcare structure and increase risk of helminth infection [11, 27]. While few of the study subjects had relied on a *curandero* for health related issues, other alternative medical personnel were more common such as *sobadores* (bone healers) and *parteras* (traditional birth attendants). Also, a large number of subjects believed ethnomedicine cures infections. Several natural remedies were reportedly used by households, and even those who did not believe these natural remedies could cure parasites did believe they served to reduce symptoms. With belief in these treatments, the incentive to seek out treatment provided in health clinics is reduced. 

Traditional gender roles in Latin American culture have been shown to play into health in spheres such as the HIV epidemic and intimate partner violence [28, 29]. These gender roles also have great implications for parasitic infections. Women may lack support in teaching children good habits, monitoring their behaviors, and maintaining clean houses as men are not required to do household chores in the *machismo* culture [12]. Husbands were active in cleaning and child caretaking in less than half of the households interviewed. If husbands played more active roles toward care of children and household cleanliness, several pathways of parasite transmission could be avoided.

The belief that cutting a child's nails causes infection is a prime example of where culture and health conflict. Dirty nails in school-age children have been shown to be associated with high-parasitic infection, and children with longer nails are able to carry more fecal infected soil, increasing the risk of infection [30, 31]. Furthermore, the belief that a hot shower after a cold day can cause infection, a remnant of the humoral theory of illness, promotes infrequent bathing which propagates poor hygiene in general. By addressing health demoting beliefs through culturally sensitive workshops, more hygienic practices can be instated in rural communities. For instance, a program aimed at eliminating parasitic transmission in this community must be integrated with the work of *curanderos*. If a local *curandero* was to indicate to people that nail cutting does not cause tetanus or explain the transmission of infected dirt through nails, individuals may be more willing to modify their practices.

### 4.3. Knowledge and Information Dissemination

Although there was a widespread awareness of the term “parasite,” many women reported having seen a parasite in their yard. This suggests that there is a general lack of knowledge regarding what a parasite actually is. Perhaps more caution will be taken if people were educated on the microscopic nature of parasites. A majority of interviewees had reported having some previous education with regards to parasites. Nonetheless, the quality and relevance of the information that is relayed are imperative to changing attitudes and behaviors. Information dissemination needs to become more widespread and this may be done if the *brigadistas* have a consistent and more frequent schedule of home visits.

The most commonly reported symptoms (diarrhea, fever, vomiting, and stomachache) were all symptoms that are associated with other common health maladies and not necessarily parasites. Symptoms that were less reported, such as anemia, poor growth, and difficulty in school, are ones that were less known and less readily recalled. If educational campaigns were to focus on these symptoms, the perceived risk presented by parasites could be increased and could result in more attention to hygienic behaviors. Only roughly half of participants understood that eating uncooked meat, defecating outdoors, or careless behavior with animals were facilitators of transmission. Campaigns should highlight these behaviors to spread more awareness in areas that are less wellknown.

### 4.4. Interaction of Poverty and Culture

As demonstrated in the correlation analysis, access to resources such as previous information did correlate with several health-promoting decisions and behaviors. Since education was linked to practice, further educational campaigns will be beneficial for this community. However, in some cases, such as the continued practice of children walking barefoot even after availability of shoes, access to resources did not result in good practices and may be more intertwined with culture.

Lastly, our study revealed the fundamental importance of filter distribution oversight and enforcement of *brigadista* visits. When *brigadistas* arevisited regularly, the attitude toward filters and use of filters was much more positive. Access to health workers did suggest improved practice and attitudes, a promising finding that indicates that local attitudes and the culture of hygiene can be improved in rural communities with proper interventions.

### 4.5. Study Limitations

Our study had a number of limitations. In our analysis of the various correlations, we selectively reported the correlations which were significant, omitting some correlations that were not significant that one may otherwise expect to show significance. For instance, access to information about parasites did not correlate with reduced misperceptions about parasites. However, these were far outnumbered by correlations that we expected and found to be present. Moreover, our study did not correlate the interacting spheres with household infection status. However, we did not feel that this was a significant concern, particularly because we were not interested in redetermining predictors of parasitic infections on which there is already substantial literature. Additionally, because of the use of multiple interviewers, we could not control for interviewer bias in the administration of the questionnaires. Nonetheless, we attempted to control for this as best as possible through interview training workshops prior to the start of the study. Furthermore, we felt that using interviewers who were members of the communities themselves may provide more unbiased data and allow interviewees to feel more comfortable.

## 5. Conclusions

Due to the complexity and multiplicity of parasitic transmission, both treatment and prevention must be coupled with educational campaigns, development and antipoverty projects, and cultural integration. Our correlational results have highlighted the ways in which several spheres that are known to predict parasitic infections interact with one another, particularly poverty and culture. While it is known that unidimensional interventions will not be wholly effective against parasites, this study further elucidates the interactions of the multiple dimensions that cause parasitic infection and provides recommendations which are likely to create effective interventions.

 While BioSand water filters will clean contaminated water, they will not prevent animals from defecating in soil that will then infect a child's nails. Nor will they convince a mother that cutting a child's nails does not cause tetanus. Providing necessary resources such as anti-parasitic medication, water filters, and antibacterial soap is only one aspect of a complete solution. Even with resources, communities may retain improper practice due to belief structures, retained behavioral practices, and poor awareness of disease transmission. Thus, social-science-focused research studies such as ours are critical in improving the control and treatment of parasites and other NTDs. By examining several areas of interest in both the hard and soft sciences surrounding the process of infection and re-infection, a better approach to disease control can be tailored.

## Figures and Tables

**Table 1:** Access to resources in communities.

Resource	Characteristic	Mean value ± SD/percentage
Water filter	Installed (*n* = 212)	28%
	Functioning (*n* = 59)	76%
Dirt floor	Present in household (*n* = 212)	65%
Well	Present on property (*n* = 211)	51%
	Distance from latrine (m) (*n* = 120)	52 ± 57%
	More elevated than latrine (*n* = 139)	45%
Latrine	Present on property (*n* = 209)	86%
Soap	Present in household (*n* = 199)	97%
*Brigadista*	Days since last visit (*n* = 84)	76 ± 97%
Anti-parasite strategy	Have received information (*n* = 205)	61%
	Treated children for parasites (*n* = 210)	88%
Health center	Distance (km) (*n* = 210)	8.3 ± 3.5%
	Days since last visit (*n* = 184)	46 ± 57%
Fencing	Animals running free (*n* = 212)	81%

**Table 2:** Knowledge, attitude, and practice regarding general hygiene and parasites.

Category	Question	Percentage (%)
Perceptions of general hygiene	Well water clean (*n* = 174)	87
	Sufficient to wash without soap (*n* = 208)	5
	Animals free of infection (*n* = 190)	13
	Dirt in yard clean (*n* = 208)	20

Attitude toward water filters	Desire to have filter (*n* = 161)	75
	Faith in efficacy of filter (*n* = 205)	80

Attitude toward hygiene	Believe they have good hygiene (*n* = 204)	92

	Parasites (*n* = 213)	85
Awareness about parasites	Worms (*n* = 213)	83
	Ameba (*n* = 213)	72

	Infection (*n* = 213)	27
	Bacteria (*n* = 213)	53
Perception of parasites	Virus (*n* = 213)	21
	Animal (*n* = 213)	50
	Have seen a parasite in yard (*n* = 207)	40

	Malnutrition (*n* = 144)	33
	Lack of energy (*n* = 144)	30
	Loss of appetite (*n* = 144)	68
	Poor growth (*n* = 144)	32
	Difficulty in school (*n* = 144)	9
	Headache (*n* = 144)	47
	Dizziness (*n* = 144)	48
Health outcomes^∗^	Anal itch (*n* = 144)	51
	Diarrhea (*n* = 144)	78
	Bloated stomach (*n* = 144)	58
	Stomach ache (*n* = 144)	80
	Anemia (*n* = 144)	19
	Fever (*n* = 144)	73
	Vomiting (*n* = 144)	81
	Death (*n* = 144)	38

	Drink dirty water (*n* = 103)	87
	Walk barefoot (*n* = 103)	83
	Eating with dirty hands (*n* = 103)	81
Transmission/high risk behaviors^∗^	Eat uncooked meat (*n* = 103)	55
	Defecating outdoors (*n* = 103)	57
	Leaving animals unfenced (*n* = 103)	41
	Washing food with dirty water (*n* = 103)	70
	Eating after touching animals (*n* = 103)	50

Perceived threat	Cause of death (*n* = 205)	98

Attitude toward parasites	Afraid of parasites (*n* = 208)	91

	Children barefoot (observed) (*n* = 209)	34
	Children barefoot (self-reported) (*n* = 208)	51
Practice of bad habits	Children without diaper (self-reported) (*n* = 174)	19
	Home-grow vegetables (*n* = 206)	39
	Well water to wash vegetables (*n* = 171)	87
	Well water to cook food (*n* = 176)	88

	Before preparing food (*n* = 100)	96
	Before eating (*n* = 100)	71
Knowledge regarding time of hand washing^∗^	After using bathroom (*n* = 100)	94
	After changing baby's diaper (*n* = 100)	54
	After cleaning yard (*n* = 100)	54
	After touching animals (*n* = 100)	59

^∗^ The “*N*” values for these questions are significantly lower than 213 due to interviewer error with the manner in which questions were asked to participants. These interviews were not counted in the data calculations.

**Table 3:** Cultural factors associated with parasites.

Category	Question	Percentage (%)
General practices	Husband help in household (*n* = 208)	46

	Cutting a child's nails causes sickness (*n* = 203)	15
Local beliefs	Cold shower after hot day causes illness (*n* = 207)	86
	Everyone always has parasites (*n* = 208)	92

Influence of local healers	*Curandero* (*n* = 212)	10
	Faith in healers (*n* = 188)	77

	Parsley (*n* = 213)	62
	Garlic (*n* = 213)	77
Natural anthelminthic	Wormseed (apasotes) (*n* = 213)	61
	Lemon water (*n* = 213)	44
	Guava leaf (*n* = 213)	56
	Faith in natural therapies (*n* = 206)	67

**Table 4:** Correlations among access to resources variables.

Variable *X*	Variable *Y*	*R* value	*P* value	*N*
	Believe well water is clean	−0.16	0.04	168
	Animals running free on property	−0.28	0.0001	204
Had previous information regarding parasites	Children barefoot at the time of interview	−0.30	0.0001	202
	Distance to the closest clinic	−0.33	0.0001	203
	Children treated for parasites	0.19	0.01	204

	Children treated for parasites	−0.18	0.01	208
Distance to the closest clinic	Number of days since last visit to the clinic	0.16	0.02	181
	Animals running free on property	0.19	0.01	210
	Presence of dirt floor	0.16	0.02	210

	Want a water filter	−0.67	0.0001	48
Number of days since last *brigadista *visit	Water filter working	−0.38	0.01	44
	Believe filter actually works	−0.51	0.0001	78

Presence of dirt floor	Education level of mother	−0.15	0.04	201

**Table 5:** Correlations among knowledge, attitude, and practice variables.

Variable *X*	Variable *Y*	*R* value	*P* value	*N*
Believe that yards are absent of infections	Believe it is sufficient to wash hands without soap	0.17	0.02	203
	Believe that animals are absent of infections	0.38	0.0001	188

Believe that parasites are animals	Claim to have seen parasite in yard	0.21	0.002	207

	Had soap in house	0.32	0.0001	190
Have good hygiene (self-reported)	Husband helps in household	0.14	0.06	199
	Latrine present	0.15	0.04	200

Children barefoot at the time of the interview	Number of children in a household	0.25	0.0003	209
	Children sometimes not wearing diapers (self-reported)	0.27	0.0005	171

**Table 6:** Correlations among cultural variables^∗^.

Variable *X*	Variable *Y*	*R* value	*P* value	*N*
People who see *curanderos* (traditional healers)	Use any traditional medicine	0.29	0.0001	212
Believe in traditional medicine	Cutting a child's nails causes sickness	0.15	0.04	179
	Believe a cold shower on a hot day causes illness	0.29	0.0001	188
Believe traditional medicines cure parasites	Believe in traditional medicines	0.28	0.0001	185
	Use any traditional medicine	0.40	0.0001	206
Husband helps in household	Children barefoot at the time of the interview	−0.15	0.03	204

^∗^ The five traditional medicinal items (garlic, lemon, water, worm seed, and guava leaf) were intercorrelated with Chronbach's *α* = 0.76.

## Appendices

### A. Pilot Study Questionnaire

#### A.1. Background Knowledge

Asking mother.

do you know what parasites are?
do you believe that parasites are a problem?
  if so, do you know how to treat parasites?
do you know how parasites are spread?
do you know the health problems parasites cause?

#### A.2. Transmission Practices

Where do you usually get water for day to day tasks, washing, drinking, and so forth?
Do you use filtered water for drinking?
  If not, why?
    do not want to (know that I should).
    do not have a filter.
    do not know it is necessary.
    other.
    do not want to (know that I should).
    do not have cooking appliances.
    do not know it is necessary.
    other.
How do you cook food?
Do you cook all the food you eat thoroughly, such as vegetables and meat?
  If not, why?
    do not want to (know that I should).
    do not have cooking appliances.
    do not know it is necessary.
    other.

*Asking about Children*.

Where is it most comfortable to go to the bathroom? Most clean?
Does your child defecate outdoors?
  Why?
    he/she wants to (knows not to).
    lack of toilet.
    do not know there are problems with defecating outside.
    other.
Are shoes really important?
Does your child walk barefoot outside?
   If so, why?
    does not want to wear shoes (but knows he should).
    he/she does not have shoes.
    does not know he should.
    other.
What do you think about washing hands?
Does your child wash his/her hands with soap after defecating and before eating?
   If not, why?
    does not want to (but knows he should).
    we do not have soap.
    does not know he should.
    other.
Do you think your child has any “dirty” habits?
Does your child eat dirt?
  does not want to (but knows he should).
  we do not have soap.
  does not know he should.
  other.

#### A.3. Additional Questions

Is hygiene important?
Where does the water you drink come from?
Is the water here clean to bathe in? Wash clothes? Wash food like fruits and vegetables? Drink?
What are some examples of good sanitation? What are some ways to clean the water?
Are there functioning toilets in the houses? Do most houses have toilets and baths?
Are the fruits and vegetables home-grown for the most part?
Are stomach pains common among children here?
What do you think causes these stomach pains?
What is the cause of health illnesses in general?
Have you heard of worms or parasites?
  What do you think regarding intestinal parasites?
What do you think regarding worms?
  Are they good or bad for health?
What are parasites and do they affect health in any way?
Where do you usually go for treatment of general illness?
Do you have traditional therapies with natural products?
How do worms/parasites live or grow?
What are shoes useful for?
What are your thoughts on defecating outdoors?

### B. Study Questionnaire

Name of the community.

*Note the following.*

Animals running free around yard? Yes/No
What kind of animals?
Is the floor of the house made of dirt? Yes/No
Were any kids not wearing shoes/sandals? Yes/No
Mothers who have (ages 1–10)
Age of mother.
Years of education of mother.
Number of children (ages 1–10).
Age of children (1–10).
How far is the closest health center?
When was the last time you or family member went?
Does your husband help with cleaning in the house? Yes/No
Many people claim their wells are clean; can I drink water from your well is it clean? Yes/No
Do you use well water directly to
  Wash food? Yes/No
  Cook food? Yes/No
We all forget to wash our hands sometimes; when do you usually wash your hands? (circle all that they say)
  Before preparing food
  After preparing food
  Before eating food
  After using the latrine
  After changing baby's diapers
  After cleaning the house/yard
  After touching the animals
  Other
Sometimes we do not have soap; does washing hands with water alone still clean enough? Yes/No
Do you have antibacterial soap or do you use regular dish-washing soap at home? AB/DW
Babies often are more comfortable without diapers; do your babies always wear diapers or not? Yes/No
Some animals keep clean while others are dirty and have infection; are your animals clean? Yes/No
Some families here say they clean their dirt and yards; do you think your dirt is clean? Yes/No
Do you grow your own vegetables here? Yes/No
Wearing shoes can often be uncomfortable; Do your children like to wear shoes/sandals or do they like to be barefoot?
  With shoes
  Without shoes
Is there a water filter installed here? Yes/No
  (if no, go to next question)
Is the filter working properly? Yes/No
Some families like filters and others do not; do you want a filter here? Yes/No
  If no, why not?
Do you think the filter actually cleans the water better? Yes/No
When was the last time a brigadista came by?
There is a belief that cutting a child's nails can cause tetanus or other infection; do you believe this is true? Yes/No
There is a belief that a cold shower after a hot day can cause sickness like fever; do you believe this is true? Yes/No
Do you see a sobadore/curandero/partera?
Do you believe this type of medicine works? Yes/No
Do you feel you have good hygiene in the house? Yes/No

#### B.1. Parasites

Do you think parasites are Sickness/Bacteria/Virus/Animal/Other?
Have you or anyone you know ever seen parasites? Yes/No
Have you ever been educated about parasites? Yes/No
People say that everyone always has parasites; do you think this is true? Yes/No
We know that parasites are bad for health; do you think they can kill people directly? Yes/No
Do you use hierba buena/ajo/agua de limón/apasotes/ojo de guayava/other?
  Can these cure parasites? Yes/No
Have you ever treated your children for parasites? Yes/No

There are many symptoms of sickness our children have. List all the symptoms or bad health effects you know that parasites cause (interviewer circle the ones subject lists): Malnutrition (they eat what I eat) /Lack of energy/Lack of appetite/Poor growth/Difficulty in school/Pain in head/Dizziness/Anal itch/Diarrhea/Expanded belly Pain in stomach/Anemia/Vomiting/Death/Others

There are many ways to get parasites. List how you think parasites are transmitted (interviewer circle the ones subject lists): Drinking dirty water/Walking barefoot/Eating with dirty hands/Eating meat that is poorly cooked/Pooping outside in the air/Letting animals roam around/Washing food with dirty water/Eating after touching animals/Others
